# Supplementary material for: Association of violence with urban points of interest
Source: PLoS One. 2020 Sep 24;15(9):e0239840. doi: 10.1371/journal.pone.0239840 (PMC7514026; doi:10.1371/journal.pone.0239840)
Supplement: S1 Appendix — (PDF) [file pone.0239840.s001.pdf]

Appendix 1: City-specific model coefficients

POI Name	Coefficient
Fast Food and Takeaway Outlets	0.1395
Nightclubs	0.1005
Convenience Stores and Independent Supermarkets	0.0771
Bus Stops	0.0648
Counselling and Advice Services	0.0583
Entertainment Services	0.0493
Estate and Property Management	0.0454
Wifi Hotspots	0.0359
Pubs, Bars and Inns	0.0046
Charitable Organisations	0.0002

Table 1: City specific model coefficients, Bristol

POI Name	Coefficient
Nightclubs	0.1929
Bus Stops	0.1425
Taxi Ranks	0.134
Pubs, Bars and Inns	0.1053
Fast Food and Takeaway Outlets	0.0735
Bookmakers	0.0582
Hotels, Motels, Country Houses and Inns	0.0264
Cash Machines	0.0143
Cafes, Snack Bars and Tea Rooms	0.0138
Historic and Ceremonial Structures	0.0054

Table 2: City specific model coefficients, Manchester

POI Name	Coefficient
Pubs, Bars and Inns	0.3246
Banks and Building Societies	0.1413
PayPoint Locations	0.0424
Bookmakers	0.0308
Hotels, Motels, Country Houses and Inns	0.0137
Vehicle Repair, Testing and Servicing	0.0084
Nightclubs	0.0082
Solicitors, Advocates and Notaries Public	0.0014
Cosmetics, Toiletries, Perfumes and Hairdressing Supplies	-0.0003
Property Sales	-0.0245

Table 3: City specific model coefficients, Cardiff

POI Name	Coefficient
Pubs, Bars and Inns	0.2646
Nightclubs	0.2147
Hair and Beauty Services	0.0437
Telephones and Telephone Cards	0.041
Clothing	0.0182
Hobby, Sports and Pastime Products	0.0177
Restaurants	0.0151
Charitable Organisations	0.0121
Container and Storage	-0.0008
Insurers and Support Activities	-0.0298

Table 4: City specific model coefficients, Leeds

POI Name	Coefficient
Bus Stops	0.1632
Convenience Stores and Independent Supermarkets	0.1444
Restaurants	0.059
Pubs, Bars and Inns	0.0571
Hair and Beauty Services	0.0309
Unspecified and Other Attractions	0.0245
Headquarters, Administration and Central Offices	0.0191
Letter Boxes	0.0089
Vehicle Repair, Testing and Servicing	-0.0002
Business Parks and Industrial Estates	-0.0032

Table 5: City specific model coefficients, Bradford

POI Name	Coefficient
Pubs, Bars and Inns	0.1987
Fast Food and Takeaway Outlets	0.1302
Nightclubs	0.1302
Counselling and Advice Services	0.0352
Convenience Stores and Independent Supermarkets	0.0264
Hobby, Sports and Pastime Products	0.0186
Restaurants	0.0094
Cash Machines	0.0048
Bakeries	0.0044
Subways	0.0004

Table 6: City specific model coefficients, Birmingham

POI Name	Coefficient
Pubs, Bars and Inns	0.4839
Bus Stops	0.1452
Convenience Stores and Independent Supermarkets	0.1144
Counselling and Advice Services	0.1
Clothing	0.0681
Parking	0.0234
PayPoint Locations	0.0021
Artists, Illustrators and Calligraphers	-0.0001
Architectural and Building-Related Consultants	-0.0254
Property Letting	-0.0503

Table 7: City specific model coefficients, Sheffield

POI Name	Coefficient
Pubs, Bars and Inns	0.4167
Cash Machines	0.1206
Nightclubs	0.0966
Cafes, Snack Bars and Tea Rooms	0.0367
Bakeries	0.0227
Bookmakers	0.0115
Charitable Organisations	0.0073
Fast Food and Takeaway Outlets	0.0064
Photographic Services	0.0021
Insurers and Support Activities	-0.0048

Table 8: City specific model coefficients, Liverpool

POI Name	Coefficient
Bus Stops	0.1224
Cash Machines	0.1051
Fast Food and Takeaway Outlets	0.0977
Employment Agencies	0.0748
Pubs, Bars and Inns	0.0523
Hair and Beauty Services	0.0408
Unspecified and Other Attractions	0.0233
Parking	0.0161
Charitable Organisations	0.0066
Estate and Property Management	0.0008

Table 9: City specific model coefficients, Leicester

POI Name	Coefficient
Pubs, Bars and Inns	0.1586
Unspecified and Other Attractions	0.1507
Bus Stops	0.1072
Charitable Organisations	0.107
Fast Food and Takeaway Outlets	0.0727
Parking	0.0529
Convenience Stores and Independent Supermarkets	0.0039
Signalling Facilities	-0.0005
Diy and Home Improvement	-0.0039
Employment Agencies	-0.0496

Table 10: City specific model coefficients, Wakefield
