# Supplementary material for: Association of violence with urban points of interest
Source: PLoS One. 2020 Sep 24;15(9):e0239840. doi: 10.1371/journal.pone.0239840 (PMC7514026; doi:10.1371/journal.pone.0239840)
Supplement: S2 Appendix — (PDF) [file pone.0239840.s002.pdf]

Appendix 2: Crime Types

Crime Type
Anti-social behaviour
Bicycle theft
Burglary
Criminal damage and arson
Drugs
Other crime
Other theft
Possession of weapons
Public order
Robbery
Shoplifting
Theft from the person
Vehicle crime
Violence and sexual offences

Table 1: Crime types, *Police.uk* open data
