# Supplementary material for: Association of violence with urban points of interest
Source: PLoS One. 2020 Sep 24;15(9):e0239840. doi: 10.1371/journal.pone.0239840 (PMC7514026; doi:10.1371/journal.pone.0239840)
Supplement: Attachment — Submitted filename: response_letter.pdf [file pone.0239840.s003.pdf]

May 2, 2020

Dear Editor,

Thank you to both yourself and the reviewers for your feedback on our submission, entitled "Association of violence with urban points of interest". We are glad the reviewers felt that this was an interesting topic. The comments were very useful and constructive, and have resulted in what we believe is a significantly better piece of work.

I will now address the comments raised by the reviewers, and highlight changes made (where applicable). Line numbers refer to those of the new, non-diffed paper.

Firstly, **Reviewer 1**:

> 1. Equation 2 only describes one type of POI. There are N crime locations ($\vec{x_1}$ to $\vec{x_N}$), M locations of point interests ($\vec{p_1}$ to $\vec{p_M}$). However, the equation should also include different types of POIs, such as pubs, bus stops, supermarkets, etc., and the set of locations and weights are different for each type of POI.

We agree that this may not have been clear enough, and have updated the text to clarify (L186).

Under this equation, the point of interest type is not considered – here we show a model that assumes each POI (regardless of type) has its own weight. We then go on to state that we apply the same weight to each POI type (L188-189).

> 2. The selection of the penalty term in Lasso regression should have theoretical justification. On page 6, it is stated that "We determine a value of α such that ten points of interest are selected". Why only ten, given that there are 619 classes of POIs. Typically, for Lasso regression, one first determines a range for the penalty parameter, with the maximum being the smallest value such that no variable is selected. A grid of the penalty parameter is then constructed. The penalty term can be determined based on cross validation, looping over the possible grid points. One may also select the penalty term using plugin method based on theoretical justifications. The authors should provide some justification on the selection of the penalty term."

The number of predictors was limited to 10 arbitrarily, as a means of concisely summarising the N most important predictors across several cities.

For completeness, we have added an additional figure showing how α effects both R^2 score and variable count (Figure 7).

> 3. The paper considers the central 3kmx3km of cities, and density is estimated using univariate kernel density estimation. Note that for points on the boundary, only data points on one side are observed, which can lead to bias in density estimations. A solution may be to use a larger grid for density estimation and use the density estimates on a smaller grid to do the Lasso regressions."

We agree that the boundary condition should have been accounted for, and have now applied padding to the sample area.

We re-calculated our results and have updated the text (L199-200) to clarify this process. We also used this as an opportunity to adjust number of folds (K) to 2, resulting in a more stable model (the addition of padding highlighted this issue).

> 4. The candidate variables in Lasso regression can include interactions between different classes of POIs. For example, the risk of crime may be high if alcohol outlets are mixed with

cash machines in close distance."

We agree that these interactions may result in a stronger predictive model, however believe that it may be outside the scope of this particular study. We have updated the future work section to address this (L338), and plan to examine interactions as part of our next work.

5. On the first line on page 6, it is stated that "vecy represents the ground-truth vectorized crime density map". I thought ground truth crime locations are only available for South Wales? (page 3, line 83)"

Reviewer 1 is correct, we have now clarified this (L220).

6. How are the cities in the study selected? Some big cities are missing, such as London, Nottingham and Glasgow. The performance of the method in cities with more complicated structures is worth investigating.

We agree that discussion around this was lacking.

Our study considers the 10 largest cities in England and Wales (excluding London, as we consider this an outlier).

According to the statistics, Nottingham is not in the top 10 most populated cities in the UK (as it has a population of 305680), so was excluded from the analysis.

Due to the population and scale of London, and the fact that we could not adequately define a "center" for the purposes of the study, we considered it an anomalous point so excluded it.

Scotland was excluded from the study as the same *Police.uk* crime data is not published by Police Scotland, making a like-for-like analysis impossible.

We have updated the text to clarify/state these points (L166-169)

7. Figure 10 (page 8) is missing from the manuscript."

We have chosen to remove this figure; we believe a tabular representation is clearest (included in appendix)

8. The combined model performance (table 6) is worse than the baseline alcohol-outlet only model (table 3) for Liverpool. Some discussions can help clarify the issue."

Having recalculated our results with padding and $K = 2$, this is no longer the case for Liverpool – however, it is for Sheffield and Wakefield. We have added some discussion around this (L316-319).

Secondly, **Reviewer 2**:

1. It would be of interest to describe what are the 15 types of crimes the authors are referring to. Considering that the authors investigate the relationship between violent crime and points of interest, are all 15 types of crimes belong to the category of violent crime? Or some of them may belong to non-violent crimes (e.g. property crimes (such as theft), bribery, etc.)?

This should have been made clearer. *Police.uk* crime data includes other crime types, such as burglary, shoplifting and drugs-related incidents, which are not necessarily violent. We limit our analysis to one of these crime types, "Violence and Sexual offences". We have updated the wording to make this clearer (L137-138), and have included a full list of crime types as an appendix.

We have also rectified the crime category type count – we had previously referenced 15 crime types, but this erroneously included the pseudo-category "All crime".

2. Please give more details on how the authors get the ground-truth vectorized crime density

map (y) and the basis matrix (B) since the definitions of them are missing. How the density of the violent crime was calculated at each point on the map from the locations of the offense in the open police data? I know that Eq (3) is based on the assumption that POIs within each category have the same weight. Eq(4) is the LASSO regression on the POI classes. Please give out the detailed derivation of the basis matrix (B) in Equation (4). It would be useful to give out the relationship of Eq(3) to the basis matrix (B) in the derivation.

We have now attempted to clarify that the basis matrix B is composed of sampled POI kernel density estimates, and hope that this clarifies the process (L223-226).

3. What are the values of the R-squared (in both Baseline model and Proposed model) for the example model output in Fig 9? Does higher R-squared always indicate a better prediction power in the Lasso based model (a higher level of similarity between Lasso predication and ground truth crime density) in authors' results?

R^2 values for the Figure 9 are listed in Table 3.

We have attempted to clarify that R^2 is indicative of better model performance (L275-277)

4. Definition is missing for R-squared score(s) authors put on line 207. How authors calculate the R-squared score? From Table 9, is R-squared score the same as the R-squared mean?

As above, we have now better defined R^2 (L275-277).

As for R^2 mean and standard deviation, we agree that this should be more clearly defined, and have done so in the revised paper (Table 3 caption)

5. "with R2 scores ranging from 0.51 to 0.70"
If R-squared scores are defined as the same as R-squared mean in the authors' manuscript, then the R-squared mean for the proposed model ranges from 0.52 to 0.65 (not from 0.51 to 0.70) based on the results from table 3. Where does this range '0.51 - 0.70' come from?

Reviewer 2 is correct, we had inadvertantly reported the range over the folds, rather than range of the mean of the folds. This has now been corrected (L280).

6. On page 8, Figure 10 missing
Figure 10 is missing. Please add figure 10.

As above – we have now chosen to remove what was figure 10, as we believe tabular formatting is the clearest representation.

7. It is an interesting idea to utilize the density of points of interest in cities to study the violent crime density and to use the lasso regression to select the most important POI classes for each city. However, the violent crime density can be closely related to community factors of neighborhoods in a city (e.g. education level, median income level, etc). Various levels of community factors can play an important role on the quality of a POI. Due to this fact, the quality of each POI in the same POI class can still be very different from each other. This fact could be an important reason that the performance of the combined model (utilizing the other 9 cities' information and predicating the 10th city) displayed in table 6, is worse than the model for an individual city (except Birmingham).

We fully agree that in general, models of violent crime benefit from the inclusion of census data, for the reasons you outline.

However, we are not convinced of the value gained by including this data in this specific analysis.

Firstly, our model focuses on city centres, where permenant residents typically make up a minority of occupants. Census data in our evaluation area unlikely to be reflective of a majority of visitors to city centres, and so without sigificant changes (for instance considering residential areas neighbouring the study area) risks adding confusion to the model.

Secondly, it is difficult to act upon knowledge that education level or income levels are associated with increases in violence. It is much easier to act on knowledge that, for instance, your city tends to see an increase in assaults around a particular venue type – this can be acted upon immediately, rather than requiring years (or decades) of fundemental changes to schooling and socioeconomic policy.

This is not to say that we disagree that individual points of interest within the same category may have stronger or weaker contributions to violence levels, this is definitely the case. In future work will look to integrate additional variables, such as venue opening hours, venue size and average unit pricing – but we believe that the analysis as it stands is still very useful.

Finally, **Reviewer 3**:

> 1. First, the paper is not trying to contribute to the theories, as there is no discussion about the theories on what determine violence crime and the related literature. The interpretation of the results and the effects of different types of poi businesses is lacking and discussions in relation to relevant literature is missing.

We agree that the discussion around theories of violent crime was lacking, and have now included a more extensive discussion and literature review (L23-79).

> 2. Second, the contribution to methodology is minor. Standard regression approach with variable selection is employed. The baseline model is too simple to make your main models good. The construction of poi related variables with kernel density was done a lot in the literature. Although the authors claim that one advantage of the work is to conduct the analysis at a fine spatial scale, the definition of unit of analysis is not clear to me.

This is an interdisciplinary study which discusses a new approach to relating city-centre features to crime levels.

In the context of this work, we believe that the use of standard techniques is a positive rather than a negative. We are using proven techniques to conduct a novel analysis. We found no other literature that studied such a broad range of points of interest over multiple cities. If this is not the case, and this analysis has been conducted before, then it would be useful to be provided with a reference to the work.

The use of Kernel Density Estimates for crime prediction is common in the literature, which we have cited. As for our model baseline, we believe this to be justified. For policy making, the locations of POIs such as pubs, takeaways and cash machines are something that is directly controllable through i.e. planning permission decisions. Other models typically consider census-derived variables, such as income or age, which are far harder to influence through policy decisions.

> 3. A comprehensive literature review regarding the theories on violence crime and related determining factors is needed. The current review is thin and mostly focuses on the method side.

As above, we agree that the initial literature review was too thin, and have now significantly expanded upon it, with a stronger focus on theories behind violent crime (L23-79)

> 4. The lack of details of models results and definition of variables. The dependent variable, density of crime counts, is not detailed. Coefficients of poi types for city-specific model are missing, which is critical for the explanation of why those factors vary across cities.

In hindsight, this was a definite mistake. We have now included model coefficients for each city as an

appendix (appendix 1)

Yours Sincerely,

Joseph Redfern (corresponding author)
Email: RedfernJM@cardiff.ac.uk
