# Supplementary material for: Association of violence with urban points of interest
Source: PLoS One. 2020 Sep 24;15(9):e0239840. doi: 10.1371/journal.pone.0239840 (PMC7514026; doi:10.1371/journal.pone.0239840)
Supplement: Attachment — Submitted filename: response_letter_2.pdf [file pone.0239840.s004.pdf]

September 11, 2020

Dear Editor,

Thank you for arranging the second review of our submission, entitled "Association of violence with urban points of interest". We appreciate the additional constructive comments from both reviewers, which we believe have once again resulted in an improved manuscript.

As before, I will now address the comments raised by the reviewers, and highlight changes made (where applicable). Line numbers refer to those of the new, non-diffed paper.

Firstly, **Reviewer 1**:

> In the Methods section, I think equations 1 and 2 should be dropped.
>
> The analysis begins with estimating kernel densities of crimes and each POI separately using equation 3, which has nothing to do with explaining the crime density by POIs. Then with the estimated densities, Lasso regression is performed to predict the crime density using the densities of POIs (equation 4). In this line of reasoning, there is no need to assume "POIs within each category have the same weight" (line 188, page 6). Please clarify if my interpretation is not right.

Equations 1 and 2 show a model that assumes that each point of interest is independently associated with crime levels, with each individual POI having its own weighting. In the context of a regression model, this is equivalent to having one independent variable for each point of interest. We attempted to explain (on line 187) that as a regularisation, we use the same weight for each POI within the same category – i.e. that all POIs of type "Cafe" would have weight x_{cafe}, that all POIs of type "Pub" would have weight x_{pub}. In this case, in the context of a regression model, this is equivalent to having one independent variable for each point of interest **category**.

We include equations 1 and 2 to try and justify and explain how we arrived at equation 3. Unlike the non-regularised model, Equation 3 **does** assume that POIs within the same category have the same weight in our model.

With this in mind, we believe that the inclusion of Equations 1 and 2 are justified; however we have attempted to clarify this point by updating the text (L199–201).

Secondly, **Reviewer 2**:

> However, after look into the details of the crime categories, you provided in the revision, I do not think (at least at this point) the database you use is persuasive to me, and therefore, I am recommending a further revision. First of all, you choose to use violence and sex offense. But is this category of a crime necessary to belong to violent crime? Sex offense may or may not involve violence.

We have no control over how categories are allocated to crime reports. "Violent and Sexual Offences" exists as a single stand alone category, rather than two categories "Violent" and "Sexual".

However, even if such a distinction were possible, we would be inclined to include Sexual offences in our analysis anyway. A sexual offence is a form of violence of a sexual nature, broadly defined by rape and sexual assault. It involves unwanted physical contact, on the part of the victim, and typically causes considerable physical and/or emotional harm.

We have updated the text to clarify this point (L139–142).

> Secondly, from the other crime types you provide in the revision (Table 1 in appendix 2), how can you tell the other crime category is not belong to violent crime? I think it is worth

> investigating which crime type is exactly violent which is not? At least, I can tell that Robbery belongs to violent crime and it is the type of crime that is available in the dataset, but you did not include it in your study. Are you certain that sex offense in the database is all belong to violent sex offense? I also suggest you add other violent crime categories (e.g. Robbery and other violent categories) into your data and to perform the analysis.

It is true that **certain** robberies could be considered violent acts, however, they are not necessarily violent. Our concern is that incorporating this crime type into our model may introduce as many false negatives as it would additional true positives.

The data needed to ascertain which of those robberies were violent and which were not violent is not in the public domain, so such an investigation, while interesting, is unforunately not possible.

Following your suggestions, we investigated how many crime reports were allocated the crime type"Violence and Sexual Offences" and how many were allocated the crime type "Robbery". There were $423,624$ instances of "Violence and Sexual Offences", and $18,119$ instances of "Robbery", i.e. "Violence and Sexual offences" were 25 times more common. If 50% of Robbery involved violence, exclusion of the category would represent a loss of $\approx 2\%$ under-reporting of violent incidents.

Furthermore, the motivation behind a robbery is different to that of violence. Robberies are typically planned and are acquisitive — i.e. the intention is to acquire goods (albeit under the threat of physical harm). However, other violence is typically more spontaneous and reactionary.

It is worth noting that these crime types are determined by how the crime was reported to the police. If the main concern was due to violence, the report may have been recorded as a violent crime. If the main concern was the (attempted) theft of property, the crime may have been recorded as robbery. With this in mind, we are confident that considering "Violent and Sexual incidents" alone is the right decision.

We have updated the text to justify the exclusion of robberies from our analysis (L143–146).

> In respect to the combined model, I think it is meaningless because every city is different, (such as income level, educational level). I do not find that it would be useful to use a model trained from other cities to perform predication in another city. The database you have is only about the POI in a very general way, but we know that even a restaurant, there is a high ending one, and there is so-so one, which depends on the income level of a city. I think those differences have an impact on violence.

Although we appreciate that a dedicated, city-specific model is optimal in most cases, we disagree that a combined model is meaningless.

As described in the manuscript (L307), we train the combined model on 9 cities, and evaluate against the remaining one, repeating 10 times, reporting our results. If this process gave 0 or negative R^2 values, then the model would be useless. However, we achieve $R^2 > 0.4$ in almost every case. This makes the combined model useful for areas without public crime data (for instance, in Scotland or Northern Ireland, which are not part of the Police.uk dataset). Every city **is** different, but not different enough that a combined model is useless. Taking into account the quality or clientele of individual POIs within the same category, while an interesting idea, is beyond the scope of this work.

We have updated the text to clarify the use case of the combined model (L331–336).

Thank you for considering our revised manuscript, we hope we have fully addressed and clarified the remain-

ing concerns of the reviewers.

Yours sincerely,

Joseph Redfern (corresponding author)
Email: RedfernJM@cardiff.ac.uk
